# Phonon Transport at Crystalline Si/Ge Interfaces: The Role of Interfacial Modes of Vibration

**DOI:** 10.1038/srep23139

**Published:** 2016-03-16

**Authors:** Kiarash Gordiz, Asegun Henry

**Affiliations:** 1 George W. Woodruff School of Mechanical Engineering, Georgia Institute of Technology, Atlanta GA, 30332, USA; 2 School of Materials Science and Engineering, Georgia Institute of Technology, Atlanta GA, 30332, USA.

## Abstract

We studied the modal contributions to heat conduction at crystalline Si and crystalline Ge interfaces and found that more than 15% of the interface conductance arises from less than 0.1% of the modes in the structure. Using the recently developed interface conductance modal analysis (ICMA) method along with a new complimentary methodology, we mapped the correlations between modes, which revealed that a small group of interfacial modes, which exist between 12–13 THz, exhibit extremely strong correlation with other modes in the system. It is found that these interfacial modes (e.g., modes with large eigen vectors for interfacial atoms) are enabled by the degree of anharmonicity near the interface, which is higher than in the bulk, and therefore allows this small group of modes to couple to all others. The analysis sheds light on the nature of localized vibrations at interfaces and can be enlightening for other investigations of localization.

Interfaces play a key role in the thermal behavior of nanostructures^1^,^2^. At small scales, interfaces have the potential to become the dominant resistance to heat conduction, which impedes the progress towards achieving improved performance in nano-electronics^3^, nano-optoelectronics^4^, or energy conversion devices such as multi-junction solar cells^5^,^6^. Thermal transport through interfaces is characterized by thermal interface conductance (TIC) (denoted by *G*), which is the constant of proportionality between the heat flow through the adjoining interface of two different materials (*Q*) and the temperature discontinuity (Δ*T*) that appears at the interface due to the heat flow (*Q *= *G*Δ*T*).

Atomic vibrations are exclusively responsible for heat conduction at interfaces between non-electrically conductive materials, but quantifying the contributions of individual eigen modes has been a challenge since the first experimental measurements of thermal interface resistance^7^ (e.g., the inverse of TIC). Recently Gordiz and Henry developed a new formalism termed interface conductance modal analysis (ICMA)^8^,^9^ that is able to determine the modal contributions to TIC by merging lattice dynamics (LD) methods with molecular dynamics (MD) simulations, offering access to the temperature dependent anharmonic dynamics of the eigen modes.

Until now, the dominant view of TIC has been based on phonon gas model (PGM) which describes the energy carried by phonons as impinging on the interface and then uses the concept of transmission probability to describe what fraction of their energy is transferred to the other side of the interface^10^,^11^,^12^,^13^,^14^,^15^,^16^,^17^,^18^,^19^,^20^,^21^. One of the conclusions that emerged from the initial development of ICMA^8^,^9^ was that a new set of eigen modes, different from the modes associated with the bulk of each separate material, must be used to properly describe the interfacial heat flow. Based on the degree of localization with respect to the interface, the modes were classified into four distinct classes: (1) extended, (2) partially extended, (3) isolated, and (4) interfacial modes^9^. The first reports on the localization of modes around interfaces and defects can be found in the works by Kosevcich *et al.*^22^, however they did not study the effect such modes have on the thermal transport properties of a system. The atomic vibrations for extended modes (type 1) are present throughout the entire structure (delocalized on both sides), extending from the bulk of one side through the interface to the bulk of the other. In this sense, these modes do not actually encounter the interface and behave like long wavelength propagating phonons, which are largely unobstructed by the presence of the interface^9^,^14^. For partially extended modes (type 2), all of the atoms on one side of the interface vibrate and partially extend to the other side, but the vibrations do not extend through the entirety of the other side (delocalized only on one side). In contrast, for isolated modes (type 3), the atomic vibrations are restricted to regions away from the interface, and there are no vibrations near the interface. In these modes, the vibrations on one side of the interface decay before reaching the interface. Lastly, for interfacial modes (type 4) a predominant portion of their vibrational energy is localized around the interface. The significance of using this new modal basis set is the fact that one cannot define a group velocity for many of the modes, since one can no longer define a dispersion and many modes are non-propagating. As a result, one cannot compute the contribution of localized modes with expressions based on the PGM. However, with the new ICMA method, one can readily calculate the contribution of any individual mode regardless of whether it is propagating or not, as one need not define its velocity or invoke the PGM.

Silicon-germanium (Si-Ge) is a prototypical system that has been studied extensively in the literature, largely due to its applications in thermoelectrics^23^,^24^. Amongst the extensive literature on Si/Ge interfaces, most studies have not been focused specifically on the modal contributions or do not include inelastic scattering^16^,^25^,^26^,^27^,^28^,^29^,^30^,^31^, except the recent studies by Chalopin and Volz^20^ and Murakami *et al.*^32^. Chalopin and Volz^20^ calculated the anharmonic spectral contributions to thermal transport across Si/Ge interfaces. Their results showed a significant contribution to TIC by frequencies around 14 THz. They suggested such a large contribution could be due to localized and non-dispersive interface modes^20^ and a similar observation has also been reported by Murakami *et al.*^32^. Here we reexamine heat conduction at strained-lattice matched, atomically smooth interfaces between crystalline Si-Ge structures with the ICMA method and new techniques for quantifying the mode level anharmonicity to better understand these contributions. Using the two techniques together allowed for deeper understanding of the nature of interfacial modes and ultimately provided a new framework for interpreting their contributions. The key distinction from previous work is the ability to conduct individual mode level assessments of not only TIC, but also each mode’s harmonic vs. anharmonic energy.

Using these techniques, we determined that the large contributions reported by Chalopin *et al.*^20^ and Murakami *et al.*^32^ are associated with modes that have amplified magnitudes of vibration for the atoms around the interface, but still extend through the bulk of the Si side. Furthermore, these modes only comprise <0.1% of the total number of modes, yet their contributions are quite substantial. We show that the large contribution by these interfacial modes originates from their high tendency to couple to almost all other modes of vibration in the system. In addition, we examine the mechanism underlying their ability to couple so strongly using a new formulation that allows for the calculation of mode-level harmonic and anharmonic energy distributions amongst the atoms in the system.

Here, the ICMA method is employed in equilibrium MD (EMD)^8^. The Tersoff potential^33^ is used to describe the interactions between the atoms in the system. For both Si and Ge sides, the number of unit cells along x, y, and z directions are chosen to be equal to 3, 3, and 24, respectively. The interface is a plane perpendicular to the z direction, which is parallel to the [100] crystallographic direction. Periodic boundary conditions are applied to all 3 spatial directions, and a finite time step of 0.5 fs is chosen for the MD simulations. After relaxing the structure under the isobaric-isothermal ensemble (NPT) for 1 ns at zero pressure and the canonical ensemble (NVT) for another 1 ns at *T* = 300 *K*, we simulate the structure in the microcanonical (NVE) ensemble for 10 ns during which the modal contributions to the heat flux across the interface are calculated. The heat flux contributions are saved and post processed to calculate the mode-mode heat flux correlation functions^8^. Statistical uncertainty, due to insufficient phase space averaging, has been reduced to less than 5% by considering 10 independent ensembles for each case^34^. All MD simulations were conducted using the Large-scale Atomic/Molecular Massively Parallel Simulator (LAMMPS) package^35^ and the eigen modes for each structure were determined from LD calculations using the General Utility Lattice Program (GULP)^36^. The Tersoff force routine in LAMMPS was modified to include the modal decomposition of the heat flux across the interface, which allows the modal contributions to be computed concurrently with the trajectory, which is computationally efficient.

The density of states (*DOS*) of the four classes of vibration as well as their population as the fraction of the total number of states 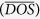 are shown in Fig. 1 and Table 1, respectively. It can be seen that the formation of the interface caused more than 2.5% of the modes to become localized near the interface, even though the entire system is crystalline and lattice matched.

Table 1 shows modal contributions to TIC associated with each class of vibration. The results are similar to Gordiz and Henry’s calculations on the TIC of LJ solids^9^ and show that interfacial modes have the highest contribution to TIC on a per mode basis (e.g., here 6.5X higher than the average contribution per mode). The accumulation function for TIC is then shown in Fig. 2a. The accumulation is interesting because it shows a steep increase between 12–13 THz. The large contribution of the modes in 12–13 THz region to TIC is both interesting and non-intuitive, because as Fig. 1 shows, it does not correspond to a region where there is a large population of modes, as would be expected for rapid increase in the accumulation at high frequencies. Similar features in the modal contributions to TIC have been observed by Chalopin and Volz^20^ and Murakami *et al.*^32^. However, using ICMA we can now pinpoint exactly which normal modes are responsible for this portion of the TIC and we can examine their characteristics to look for deeper insights.

LD of the combined structure revealed that 12–13 THz region is comprised of large contributions from six special interfacial modes, which comprise <0.1% of the all the modes, yet they are responsible for approximately 15% of the TIC (Fig. 2a). Figure 3 shows pictures of the eigen vectors associated with this small group of six modes, which indicates that many of them extend through the bulk of the Si side, but have a predominant portion of their energy/vibration (∼20%) at the interface (see supplementary note 1 on how to calculate the energy distribution of one eigen mode over different atoms in the system). Furthermore, since the frequency of these interfacial modes are above the maximum frequency of the bulk Ge side (∼10 THz) their contribution must be the result of inelastic interactions enabled by the system’s anharmonicity^8^,^9^.

Using ICMA, the degree of interaction/correlation between each pair of vibrational modes in the system can be calculated and presented as a two-dimensional map of correlation^8^,^9^, shown in Fig. 2b. Since elastic interactions are restricted to phonons of the same frequency, which are only associated with the values along the diagonal of the correlation map (Fig. 2b), all the off-diagonal contributions are attributed to the anharmonicity. The 2D mapping shows that the interfacial modes between 12–13 THz are strongly correlated with all others and their correlation is at least 1 order of magnitude larger than the average correlation outside this regime (see Fig. 2c for the 3D representation of the correlations).

Since the highly contributing modes of vibration shown in Fig. 3 have relatively large frequencies (∼12–13 THz), one could suspect their contribution to TIC at room temperature to be partially suppressed by their reduced heat capacity. However, even after such quantum effects are accounted for^9^,^37^ at T = 300 K, the interfacial modes of vibration at 12–13 THz still contribute 12% to TIC (Fig. 4b). Additionally, the modal contributions to TIC at higher temperatures (400 K and 500 K (Fig. 4a,b)) show that interfacial modes still contribute 15% to the TIC, which confirms that for applications at room-temperature and above, interfacial modes maintain their contribution to TIC.

It is also important to note that if such a large contribution to the conductance is associated with a narrow range of frequencies for a real system, such a distinct feature could potentially be verified by measuring the thermal interface conductance vs. temperature (i.e., using transient thermoreflectance techniques^38^,^39^). The data in Fig. 5 supports this idea by showing the effect of interfacial modes on the TIC accumulation function. It can be seen that by removing the entire contribution of interfacial modes (e.g., by excluding all the points corresponding to interfacial modes on the correlation maps) the sharp increase at the frequency range of 12–13 THz disappears and the TIC accumulation function follows a smooth increase vs. frequency. Moreover, the exclusion of interfacial modes results in a noticeably different temperature dependence for the TIC when quantum corrections are applied, which can form a basis for the experimental evaluation of the existence of the reported interfacial modes in the 12–13 THz frequency region.

To understand how the interfacial modes between 12–13 THz couple to virtually all other modes, we then computed the degree of anharmonicity sampled by different atoms at different locations in the structure (see Fig. 6). This was done according to the procedure presented in supplementary note 1. In essence the approach is based on the calculation of two main quantities. First, the harmonic energy attributed to an atom *i* by the n^th^ eigen mode 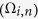 can be written as (see supplementary note 1 for the derivation),


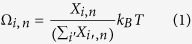


where 
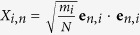
, and *N* is the total number of unit cells in the system, *m*_*i*_ is the mass of atom *i*, and 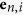 is the associated eigen vector for atom *i* participating in eigen mode *n*. As explained in the supplementary note 1, this approach is exactly equivalent to calculating the harmonic potential energy of each atom through its interactions with all other atoms, via its effective spring constant with each atom (e.g., elements of the dynamical matrix). The second quantity is the full potential energy attributed to atom *i* by the n^th^ eigen mode 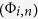, which includes both the harmonic and anharmonic portions (anharmonic to full order). This is determined by partitioning the energy of interaction 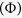 among the interacting atoms (e.g., 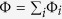), which is straightforward^40^,^41^, by simply counting all pairs twice and associating half of every pair’s energy with each atom. An atom’s full potential energy contribution to a given mode is then determined by applying the associated displacements for a specific eigen mode^42^ – e.g., the case where it is the only mode excited in the system. After applying the displacements associated with a given mode, each individual atom’s potential energy can be calculated statically, without having to execute an EMD simulation. The difference between the total 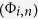 and harmonic energies 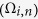 attributed to atom *i* by eigen mode *n* is then the summation of all anharmonic terms in the potential energy 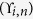,


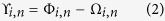


If one then sums 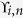 over all the eigen modes, the result is the total anharmonic energy of atom *i*, and provides insight into the amount of anharmonicity it will experience. We can then sum the anharmonic energy contributions for all of the atoms in a specific region of the system which will provide insight into whether or not the presence of the interface causes different regions of the system experience more or less anharmonicity.

Figure 6 shows that atoms near the interface experience the largest anharmonic contributions to their energy. This confirms that the presence of the interface increases the extent to which atoms access the anharmonic terms in the energy in certain regions. It should be noted, that by comparison, for a homogenous solid (no interface) the anharmonic energy terms would be uniform and homogenous throughout the entire structure. Thus, a deviation from constant anharmonicity is attributable to the presence of the interface itself. Also, since the interfacial modes hold 20% of their energy in this more anharmonic region, one might immediately assume that the motions associated with these modes must be the most strongly anharmonic. However, from the mode-level contributions to anharmonic energy for the interfacial atoms (see Eq. (2) and Fig. 7), it appears that interfacial modes between 12–13 THz are not the most notably anharmonic modes in the interfacial region. Nonetheless, they do comprise the predominant portion of the energy in the interfacial region. Figure 8b shows that more than 30% of the energy for interfacial atoms comes from the interfacial modes with frequencies between 12–13 THz. Thus, despite their small population in the entire system DOS (Figs 1 and 8a), they contribute considerably to the energy of the atoms in the interfacial region (Fig. 8b), which matter most for the TIC. We then postulated that other modes, which could be somewhat more anharmonic (see Fig. 7), tend to couple their energy from the bulk into these interfacial modes, which have the most energy in the interfacial region. These interfacial modes then facilitate energy transfer through the interface and into the other material. As a result, interfacial modes exhibit extremely strong correlation with all the other modes (Fig. 2b,c), as they effectively serve as a bridge for the energy to couple across the interface. This supports a new physical picture for describing the contributions of interfacial modes, whereby the energy in other modes couples to the most overall energetic modes in the interfacial region (e.g., interfacial modes), which then move the energy across the interface to the other material, whereby it can couple to other modes that exist in the bulk of the other side.

The scheme provided for calculating the mode-level anharmonic energy contributions from each atom in the system 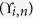 (Eq. 2) is a simple and straightforward technique to quantitatively assess the degree of anharmonicity in the interactions in essentially all classes of solid materials. It could be used, for example in crystalline solids or systems with interfaces, defects or even disordered solids. For instance, Fig. 6 not only shows that the interface region is the most anharmonic region, but it also shows that anharmonic energy in the bulk of the Ge side is on average higher than the Si side. This is interesting, because the higher anharmonic energy on the Ge side could be one of the factors that leads to the lower thermal conductivity of Ge compared to Si (i.e., lower relaxation times)^43^.

Using the ICMA method, we identified that the high contributions to TIC from modes with frequencies between 12–13 THz at the interface of crystalline Si/Ge structures are caused by a small group interfacial modes that occupy less than 0.1% of the total population of modes. These interfacial modes have extended vibrations on the bulk of Si side and have a strong tendency to couple to virtually all other modes in the system. Our calculations at T = 300 K show that at the interface ∼23% of the potential energy is attributable to the anharmonic terms in the potential energy, which leads to strong coupling between modes with different frequencies. However, this relatively large degree of anharmonicity quickly decreases further into the bulk of each side (<7% anharmonicity in the bulk). Furthermore, this degree of anharmonicity facilitates strong correlations between interfacial modes and virtually all other modes in the structure. By performing energy distribution calculations, we showed that although the interfacial modes are not the most anharmonic modes in the entire structure, but they are the most energetic modes in the most anharmonic region (e.g., the interfacial region). Consequently, all the other modes tend to couple with them to transfer their energy to the other side of the interface. These results therefore provide a basis for developing a new and revised physical picture for thinking about the contributions of localized modes at interfaces.

## Additional Information

**How to cite this article**: Gordiz, K. and Henry, A. Phonon Transport at Crystalline Si/Ge Interfaces: The Role of Interfacial Modes of Vibration. *Sci. Rep.* **6**, 23139; doi: 10.1038/srep23139 (2016).

## Supplementary Material

Supplementary Information

## Figures and Tables

**Figure 1:**
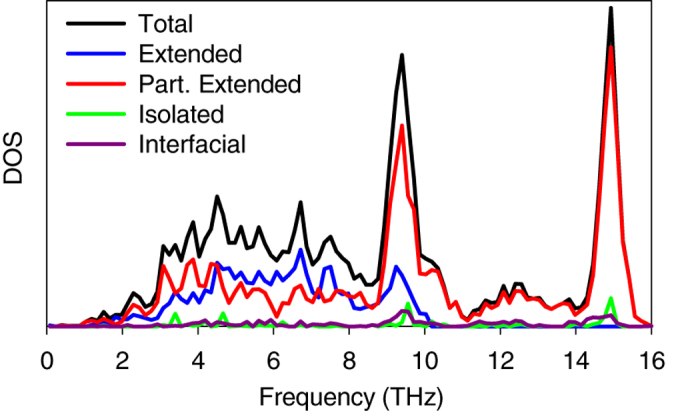
DOS of the modes of vibration across the crystalline Si/Ge interface. Summation of the DOS for different classes of vibration (colored curves) are equal to the total DOS (black curve).

**Figure 2:**
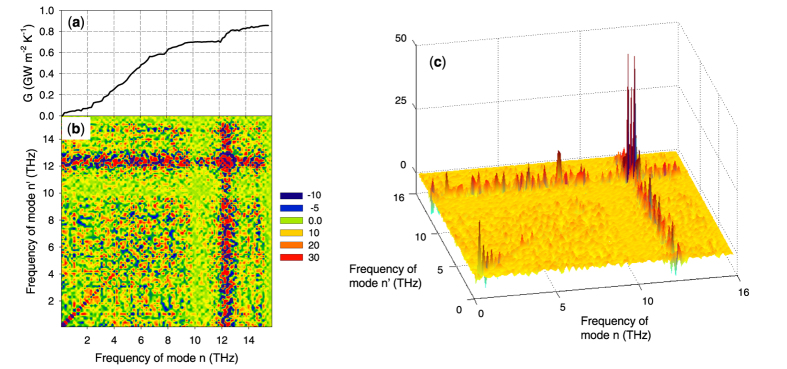
Modal contributions to TIC for Si/Ge interface at T = 300 K. (**a**) TIC accumulation function, (**b**) 2D map and (**c**) 3D perspective depiction of the data in (**b**) showing the magnitudes of the correlations/interactions as elevations above the plane of two frequency axes. The values presented on the 2D and 3D maps have units of 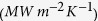. Inelastic interactions occur between the modes with frequencies 12–13 THz and all the other modes in the system. Although panel (**a**) shows that interfacial modes in the frequency range of 12–13 THz contribute almost 15% to the TIC, the summation of the contribution of interfacial modes on the correlation maps of (**b**) and (**c**) show that when their affects/correlations with other modes are also included they are, in total, responsible for more than 26% of the total TIC.

**Figure 3:**
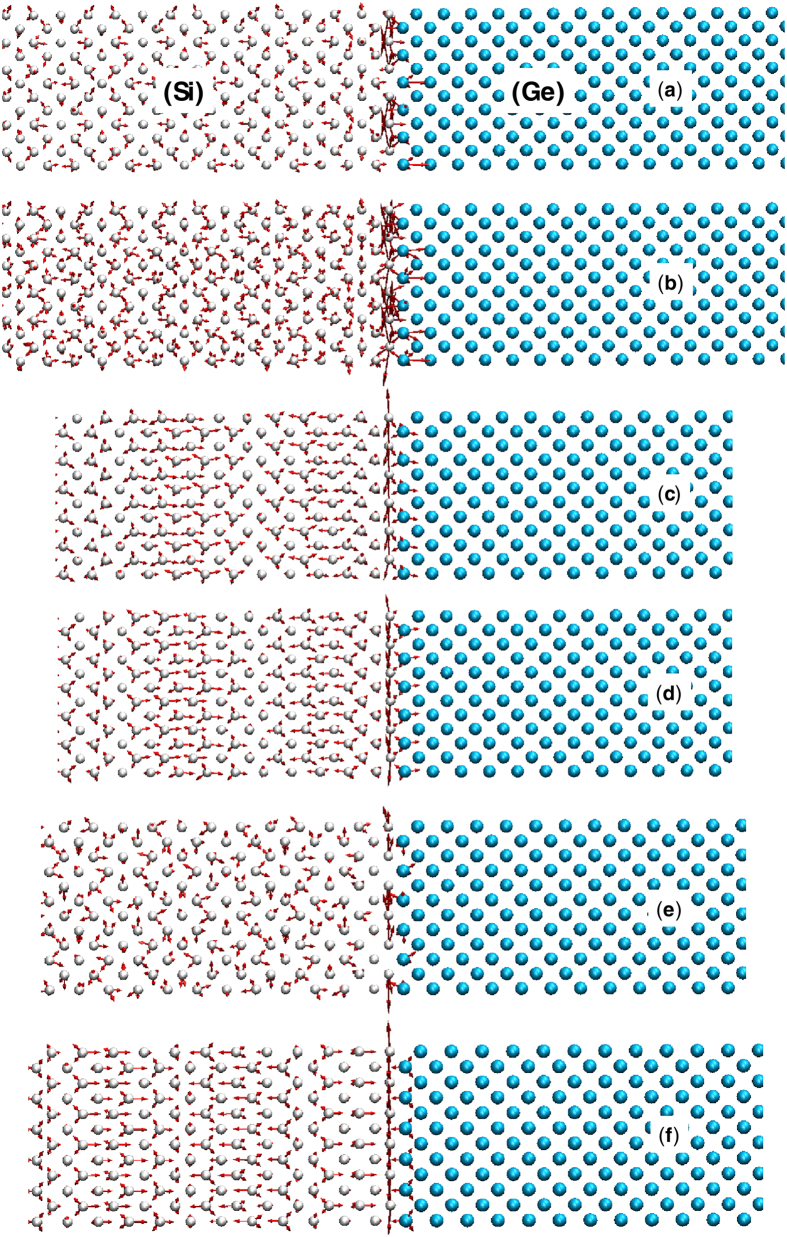
Eigen vectors for six special interfacial modes in the 12–13 THz region that comprise near 15% of the TIC. The frequencies of these eigen modes are (**a**) 12.01 THz, (**b**) 12.01 THz, (**c**) 12.10 THz, (**d**) 12.10 THz, (**e**) 12.25 THz, and (**f**) 12.32 THz. Si and Ge atoms are shown with white and cyan spheres respectively.

**Figure 4:**
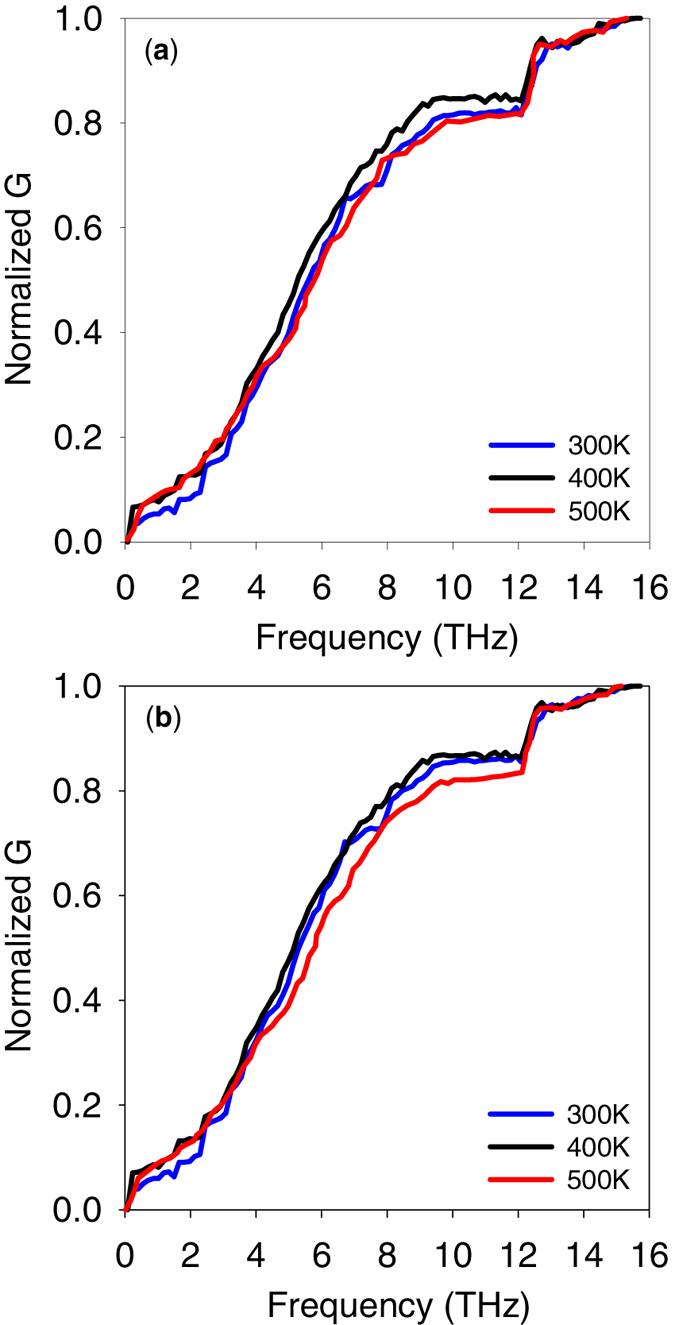
Normalized modal contributions to TIC for Si/Ge interface at three different temperatures of 300 K, 400 K, and 500 K (**a**) before quantum correction and (**b**) after quantum correction. The absolute values of TIC before quantum correction for 300 K, 400 K, and 500 K are 0.84 GWm^−2^K^−1^, 0.87GWm^−2^K^−1^ and 0.88 GWm^−2^K^−1^, respectively. The absolute values of TIC after quantum correction for 300 K, 400 K, and 500 K are 0.80 GWm^−2^K^−1^, 0.83GWm^−2^K^−1^ and 0.86 GWm^−2^K^−1^, respectively.

**Figure 5:**
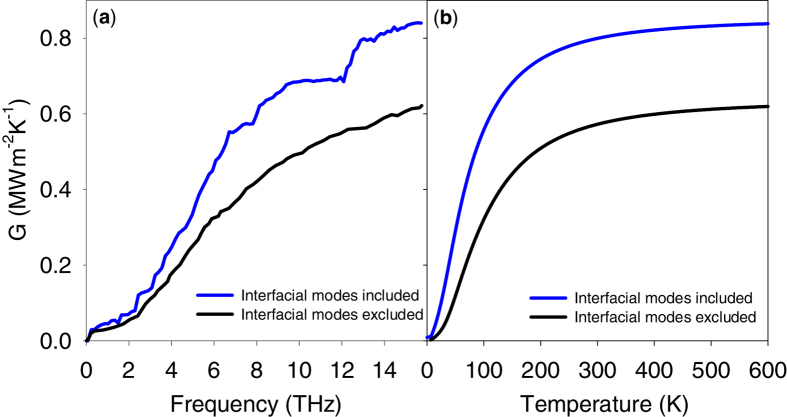
Effect of excluding the contribution by interfacial modes at the frequency range of 12–13 THz on (**a**) the TIC accumulation function and (**b**) the temperature dependence of TIC at the interface of crystalline Si/Ge. It should be noted that according to the correlation maps shown in Fig. 2, the contribution/effect by interfacial modes is not limited to the narrow frequency region of 12–13 THz and is in reality distributed all over the frequency spectrum. Transient thermoreflectance measurement techniques can potentially detect the decrease in TIC after the exclusion of interfacial modes, which can serve as an experimental basis for evaluating the existence of these modes.

**Figure 6:**
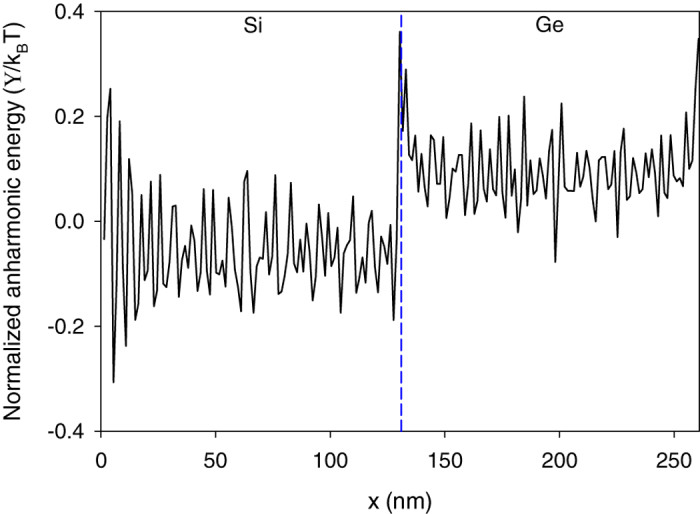
Average anharmonic energy for the atoms at each layer along the Si/Ge structure normalized by k_B_T. The largest peak belongs to the last layer of Si atoms at the interface. The position of the interface is shown by the dashed line.

**Figure 7:**
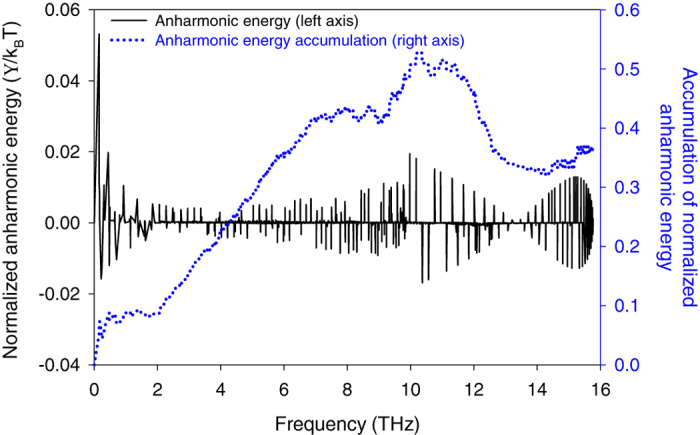
Mode-level distribution of anharmonic energy, normalized by k_B_T, averaged over the first layer of Si atoms at the interface that have Ge atoms as nearest neighbors. This layer is also the most anharmonic region in the structure (see Fig. 6). Anharmonic energy accumulation function is also presented in the figure.

**Figure 8:**
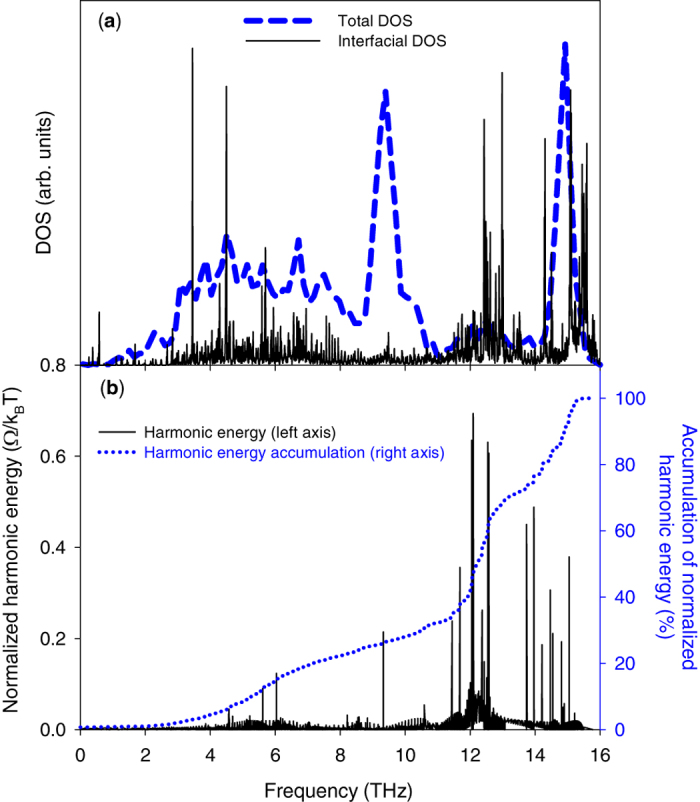
(**a**) DOS and (**b**) Mode-level distribution of harmonic energy, normalized by k_B_T, for the last layer of Si atoms at the interface, that has the largest anharmonicity (see Fig. 6) in the Si/Ge structure. Panel (**a**) also includes the DOS for the entire structure, which is equal to total DOS curve in Fig. 1. Spectral energy distribution for the interfacial atoms in panel (**a**) is determined from the MD simulated atomic velocities^44^,^45^. The difference between the DOS of the interfacial atoms and the DOS for the entire structure is significant as it appears that the optical phonon peak in Ge between 8–10 THz is shifted to 12–13 THz in the interfacial region.

**Table 1:** Number of states for the four different classes of vibration and their contribution to TIC across the Si/Ge interface.

Mode Type	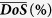	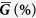	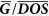
Extended	29.35	51.99	1.77
Partially extended	64.24	29.28	0.45
Isolated	3.50	<0.01	<0.01
Interfacial	2.90	18.73	6.45

Columns 2–4 represent the fraction of the total number of states 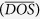, the percentage contribution to ***G*** 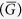, and contribution to ***G*** divided by fraction of total number of states (i.e., contribution to G per mode) 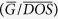, respectively.
