# Supplementary material for: Phonon Transport at Crystalline Si/Ge Interfaces: The Role of Interfacial Modes of Vibration
Source: Sci Rep. 2016 Mar 16;6:23139. doi: 10.1038/srep23139 (PMC4793224; doi:10.1038/srep23139)
Supplement: Supplementary Information [file srep23139-s1.pdf]

Title: Phonon Transport at Crystalline Si/Ge Interfaces: The Role of Interfacial Modes of Vibration

Authors: Kiarash Gordiz[1], Asegun Henry[1,2,*]

Affiliations:

[1]George W. Woodruff School of Mechanical Engineering, Georgia Institute of Technology, Atlanta GA, 30332

[2]School of Materials Science and Engineering, Georgia Institute of Technology, Atlanta GA, 30332

*Correspondence to: ase@gatech.edu

Supplementary Notes:

Supplementary Note 1. Mode-level harmonic and anharmonic energy distributions

The potential energy for an oscillator can be written as arising from the sum of harmonic and anharmonic contributions,

$$\langle H_{pot} \rangle = \langle H_{harmonic} \rangle + \langle H_{anharmonic} \rangle \quad (1)$$

where $\langle \cdots \rangle$ represents the ensemble average[1]. The anharmonic portion represents the deviation of the potential energy of the system from its largest component which is the harmonic term. We are interested in calculating how the harmonic and anharmonic portions of the energy for an oscillator are distributed amongst the different atoms in the system. Based on the equipartition theorem, the average harmonic energy of a classical oscillator is equal to,

$$\langle H_{harmonic} \rangle = \frac{1}{2} k_B T \quad (2)$$

where k_B is the Boltzmann constant, and T is the temperature of the system. The potential energy for a harmonic oscillator can also be calculated from the normal mode amplitude analysis via[2],

$$\langle H_{harmonic} \rangle = \frac{1}{2} X_n{}^2 \omega_n{}^2 \quad (3)$$

where X_n is the modal displacement coordinate, and ω_n is the frequency of the eigen mode. It should be noted that calculating the harmonic energy for an eigen mode using the knowledge of force constant matrix and the respective atomic displacements for an eigen mode is equal to the approach based on the normal mode amplitude analysis (Eq. (3)). The modal displacement coordinate can be explicitly written as [2],

$$X_n = \sum_i \sqrt{\frac{m_i}{N}} \mathbf{e}_{n,i} \cdot \mathbf{u}_i \quad (4)$$

where, N is the total number of unit cells in the system, i is the atom index, m_i is the mass of atom i, $\mathbf{e}_{n,i}$ is the eigen vector associated with atom i for eigen mode n, and $\mathbf{u}_i$ is the displacement of atom i. By combining Eq. (2) and Eq. (3) we then have,

$$X_n{}^2\omega_n{}^2 = k_B T \quad (5)$$

To determine the distribution of the harmonic energy for eigen mode n amongst the atoms in the system, one can envision a state of the system whereby all of the atoms in the system are displaced from equilibrium in the direction of their respective eigen vectors from mode n (i.e., $\mathbf{e}_{n,i}$). In this view, the attributed displacement to an atom would be equal to,

$$\mathbf{u}_i = \alpha_n \mathbf{e}_{n,i} \quad (6)$$

where α_n is a scaling factor that associates a certain degree of displacement with the mode's amplitude at a given temperature. The exact value of the scaling factor can then be calculated from the combination of Eqs. (4-6). Replacing for the atomic displacement in Eq. (4) with the definition in Eq. (6), we would have,

$$X_n = \sum_i \sqrt{\frac{m_i}{N}} \mathbf{e}_{n,i} \cdot \alpha_n \mathbf{e}_{n,i} = \alpha_n \sum_i \sqrt{\frac{m_i}{N}} \mathbf{e}_{n,i} \cdot \mathbf{e}_{n,i} \quad (7)$$

which by the substitution of $X_{i,n} = \sqrt{\frac{m_i}{N}} \mathbf{e}_{n,i} \cdot \mathbf{e}_{n,i}$, yields a simpler form,

$$X_n = \alpha_n \left(\sum_i X_{i,n} \right) \quad (8)$$

Using Eq. (5) we then have $X_n \omega_n = \sqrt{k_B T}$ and by incorporating Eq. (8), we can calculate the scaling factor as,

$$\alpha_n = \frac{\sqrt{k_B T}}{\left(\sum_i X_{i,n} \right) \cdot \omega_n} \quad (9)$$

With this scaling factor (α_n), we then rewrite Eq. (5) as,

$$X_n \; X_n \; \omega_n{}^2 = k_B T \quad (10)$$

and substitute for X_n from Eq. (8), which yields,

$$\left[\alpha_n \left(\sum_i X_{i,n} \right) \right] \left[\alpha_n \left(\sum_{i'} X_{i',n} \right) \right] \omega_n^2 = k_B T \quad (11)$$

$$\sum_i X_{i,n} \left(\alpha_n^2 \right) \left(\sum_{i'} X_{i',n} \right) \omega_n^2 = k_B T \quad (12)$$

Replacing for α_n with its definition in Eq. (9) allows us to rewrite Eq. (12) as,

$$\sum_i X_{i,n} \frac{k_B T}{\left(\sum_{i''} X_{i'',n} \right)^2 \omega_n^2} \left(\sum_{i'} X_{i',n} \right) \omega_n^2 = k_B T \quad (13)$$

and finally,

$$\sum_i \frac{X_{i,n}}{\left(\sum_{i'} X_{i',n}\right)} k_B T = k_B T \tag{14}$$

Eq. (14) defines the harmonic energy attributed to atom i by eigenmode n $\left(\Omega_{i,n}\right)$,

$$\Omega_{i,n} = \frac{X_{i,n}}{\left(\sum_{i'} X_{i',n}\right)} k_B T \tag{15}$$

Through the definition of the scaling factor $\left(\alpha_n\right)$, we can determine the exact displacements for the atoms in the system when exactly one mode is excited. This then allows us to analyze the energy and anharmonicity associated with that single mode, where (according to Eq. (2)) the harmonic energy for eigen mode n is equal to $\frac{1}{2} k_B T$.

Utilizing the exact same displacements for a singly excited mode in the system, we can evaluate the total potential energy for each atom in the system for that eigenmode $\left(\Phi_{i,n}\right)$. This is achieved by writing the total potential energy of the system as the summation over the individual atomic potential energies as[3,4]

$$\Phi = \sum_i \Phi_i \tag{16}$$

where Φ is the total potential energy and Φ_i is the potential energy assigned to atom i, such that all energy is equally partitioned amongst interacting pairs of atoms. For an eigenmode n, the difference between the total potential energy $\left(\Phi_{i,n}\right)$ and the harmonic potential energy $\left(\Omega_{i,n}\right)$ associated with atom i equals the anharmonic portion of the energy $\left(\Upsilon_{i,n}\right)$.

$$\Upsilon_{i,n} = \Phi_{i,n} - \Omega_{i,n} \tag{17}$$

This anharmonic energy can then be used to better understand how modes and regions of atoms interact and ultimately will help to quantify its effect on transport.

1 Gordiz, K., Singh, D. J. & Henry, A. Ensemble averaging vs. time averaging in molecular dynamics simulations of thermal conductivity. *Journal of Applied Physics* **117**, 045104 (2015).

2 Dove, M. T. *Introduction to lattice dynamics*. Vol. 4 (Cambridge university press, 1993).

3 Hardy, R. J. Energy-flux operator for a lattice. *Physical Review* **132**, 168 (1963).

4 Henry, A. S. & Chen, G. Spectral phonon transport properties of silicon based on molecular dynamics simulations and lattice dynamics. *Journal of Computational and Theoretical Nanoscience* **5**, 141-152 (2008).
